# Protective Effect of *Thymus serrulatus* Essential Oil on Cadmium-Induced Nephrotoxicity in Rats, through Suppression of Oxidative Stress and Downregulation of NF-κB, iNOS, and Smad2 mRNA Expression

**DOI:** 10.3390/molecules26051252

**Published:** 2021-02-26

**Authors:** Mohd Nazam Ansari, Najeeb Ur Rehman, Aman Karim, Faisal Imam, Abubaker M. Hamad

**Affiliations:** 1 Department of Pharmacology & Toxicology, College of Pharmacy, Prince Sattam Bin Abdulaziz University, Al-Kharj 11942, Saudi Arabia; 2 Department of Biological Sciences, National University of Medical Sciences, Rawalpindi 46000, Pakistan; aman.karim@numspak.edu.pk; 3 Department of Pharmacology and Toxicology, College of Pharmacy, King Saud University, Riyadh 12372, Saudi Arabia; fimam@ksu.edu.sa; 4 Department of Basic Sciences, Preparatory Year Deanship, Prince Sattam Bin Abdulaziz University, Al-Kharj 11942, Saudi Arabia; a.hamad@psau.edu.sa; 5 Department of Histopathology and Cytopathology, Faculty of Medical Laboratory Sciences, University of Gezira, Wad Madani 21111, Sudan

**Keywords:** cadmium, inflammation, NF-κB, renal injury, Smad 2, *Thymus serrulatus*

## Abstract

The purpose of the research was to examine the protective effect of essential oil from *Thymus serrulatus* Hochst. ex Benth. (TSA oil) against cadmium (Cd)-induced renal toxicity. The experimental protocol was designed using 30 healthy adult Wistar albino rats allocated into five groups containing six animals in each group. Group 1 was treated as normal control and groups 2, 3, 4, and 5 were treated with cadmium chloride (CdCl_2_, 3 mg/kg, IP) for 7 days. Group 3 was also treated with silymarin (100 mg/kg, PO) as a standard group, while groups 4 and 5 were administered with TSA oil at doses of 100 and 200 mg/kg PO, respectively. The nephrotoxicity was measured with various parameters such as kidney function markers, oxidative stress markers (glutathione (GSH) and malondialdehyde (MDA)), and messenger ribonucleic acid (mRNA) expression levels of inflammatory factors. The histological studies were also evaluated in the experimental protocol. The CdCl_2_-treated groups showed a significant increase in the levels of serum kidney function markers along with MDA levels in kidney homogenate. However, renal GSH level was found to be reduced significantly. It was found that CdCl_2_ significantly upregulated the nuclear factor levels of kappaB (NF-κB p65), inducible nitric oxide synthase (iNOS), and small mothers against decapentaplegic (Smad2) as compared to the normal control group. On the other hand, TSA oil significantly improved the increased levels of serum kidney function markers, non-enzymatic antioxidants, and lipid peroxidation. In addition, TSA oil significantly downregulated the increased expression of NF-κB p65, iNOS, and Smad2 in Cd-intoxicated rats. Moreover, the histological changes in the tissue samples of the kidney of Cd-treated groups were significantly ameliorated in the silymarin- and TSA-oil-treated groups. The present study reveals that TSA oil ameliorates Cd-induced renal injury, and it is also proposed that the observed nephroprotective effect could be due to the antioxidant potential of TSA oil and healing due to its anti-inflammatory action.

## 1. Introduction

Environmental chemical exposure remains a major public health problem globally. Exposure to cadmium (Cd), one of the most reactive toxic metals, has increased in the biosphere from both natural and anthropogenic sources [1]. The Cd exposure is also due to the contamination in soil, air, water, and food, as well as cigarette smoke [2]. A significantly increased absorption along with decreased excretion rate leads to an increased burden of Cd in different organs. The Cd can damage multiple organs depending on its dose, route, and duration but mainly affects kidneys and causes renal impairment [3].

The kidneys are vital organs in the human body and responsible for various essential functions, including the removal of harmful metabolites, nitrogenous wastes, and some drugs with urine [4]. Earlier studies reported that Cd toxicity results in irreversible dysfunction of renal tubules [5] and causes decreased removal of the toxic chemicals, drugs, or both that lead to acute kidney failure. Moreover, renal injury caused by chronic Cd exposure may lead to chronic kidney failure and if not treated can result in death [6,7]. Cd accumulation in proximal convoluted tubules obstructs tubular reabsorption and results in polyuria and proteinuria. There have been studies of common abnormal effects of Cd on kidney function, but there is still a lack of detailed information on molecular mechanisms and a need to explore this further [8].

Studies over the past few decades revealed that the maximum probability of renal damage is because of the release of Cd in the cell that provokes oxidative stress [9,10]. One of the suggested mechanisms is the disturbance in the natural antioxidant defense system, which further causes the overproduction of oxygen free radicals (OFR) [11] and results in decreased glutathione (GSH) levels and, in turn, oxidative stress-related apoptosis [12]. Out of several reported mechanisms, decreased GSH content and OFR-induced cell damage are mainly responsible for lipid peroxidation [13,14].

Previous literature also finds that the correlation between oxidative stress and inflammation enhances various renal diseases [15]. Therefore, it can be suggested that renal toxicity is caused by Cd through complex intracellular signaling pathways which are mainly directed by oxidative stress. Nuclear factor-κB (NF-κB) controls several inflammatory genes in all types of cells [16]. NF-κB is the main cause of various kidney diseases and can be activated by Cd exposure. Chronic kidney disease has been found in both human and animal models by upregulation of Smad2 proteins [9]. Earlier studies stated that downregulation of NF-κB and Smad2 might be beneficial mechanisms for the anti-inflammatory drugs [17].

Amongst the treatment policies, it is hypothesized that attenuation of Cd-induced oxidative stress using antioxidants either from natural or synthetic sources is considered as the main possible approach for the treatment of Cd-induced nephrotoxicity. Therefore, researchers have recently sought defensive mechanisms against toxicity caused by chemicals or drugs and have also considered biologically active compounds with antioxidant and inflammatory impact [18,19].

In different parts of the world, different species of the genus *Thymus* have traditionally been used to treat various disorders such as bronchitis, asthma, pertussis, laryngitis, tonsillitis, and cough [20]. *Thymus serrulatus* Hochst. Ex Benth (family Lamiaceae) grows in Ethiopia and has traditionally been used to treat influenza [21] and cough [22]. *T. serrulatus* is a much-branched perennial subshrub that grows in the Afromontane and Afroalpine zones of Ethiopia and Eritrea [23].

*T. serrulatus* has been reported to possess anthelmintic, antibacterial, fungicidal [23], diuretic [22], vasodilatory [24], and antihyperlipidemic [25] activities. Further, the essential oil of *T. serrulatus* (TSA oil) has been reported to possess antiseptic, antifungal, and vermifuge properties, among others [26]. The essential oil of the plant from different Ethiopian localities showed promising hepatoprotective activity in rats [27]. Additionally, previous studies have shown that the pharmacological activities of the essential oil are positively correlated with the presence of active ingredients such as thymol, carvacrol, p-cymene, γ-terpinene, and rosmarinic acid [26,27]. On the other hand, thymol and carvacrol, which have been found as major constituents in essential oil, are important natural products with free radical scavenging activities and antioxidant properties [28]. A previous study reported that a combination of thymol and carvacrol had a synergistic nephroprotective effect that might be attributed to antioxidant, anti-inflammatory, and antiapoptotic activities [29]. Additionally, thymol and carvacrol, which have been found as major constituents in essential-oil-containing plants, also caused a decrease in the immunoglobulin E (IgE), interleukin-4 (IL-4), interleukin-5 (IL-5), and interleukin-13 (IL-13) levels as well as the number of inflammatory cells that cause airway disorders [30].

As far as we know, the effect of *T. serrulatus* on nephrotoxicity has not been reported. Thus, in this study, we hypothesized and attempted to investigate the nephroprotective effect of the essential oil extracted from *T. serrulatus* of Ethiopian origin against Cd-induced nephrotoxicity using Wistar albino rats by evaluating kidney function markers, inflammatory markers, antioxidant status, and histological changes.

## 2. Results

### 2.1. Essential Oil Yield (%)

Hydrodistillation of the aerial part of the *T. serrulatus* gave 0.09% (*v*/*w*) pale yellow essential oil with a characteristic odor.

### 2.2. Effect of TSA Oil on Biomarkers of Kidney Function

**Figure 1:**
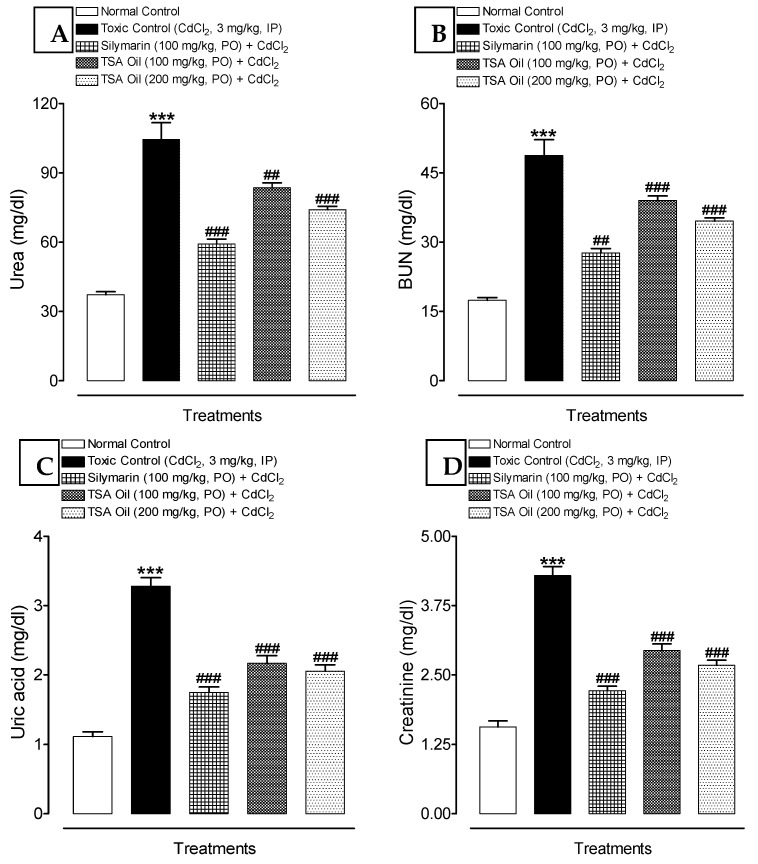
Effect of *Thymus serrulatus* Hochst. ex Benth. oil (TSA oil) on kidney function markers (**A**) urea, (**B**) blood urea nitrogen (BUN), (**C**) uric acid, and (**D**) creatinine in serum against Cd-induced renal injury in rats. *** *p* < 0.001, shows the comparison of toxic control with untreated (normal control) group (unpaired *t*-test); ^##^ *p* < 0.01 and ^###^ *p* < 0.001, show the comparison of a treated group with toxic control (one-way ANOVA followed by Tukey test). Each bar represents mean ± SEM (*n* = 6).

The levels of blood urea, uric acid, creatinine, and blood urea nitrogen (BUN) were found to be significantly elevated (*p* < 0.01) in rats that were treated with Cd only (3 mg/kg, IP) for 7 days. However, administration of TSA oil and silymarin in Cd-intoxicated rats substantially improved the Cd-induced kidney damage as specified by suppressed levels of blood urea, uric acid, creatinine, and BUN (Figure 1A–D).

### 2.3. Effect of TSA Oil on Lipid Peroxidation and Oxidative Stress

**Figure 2:**
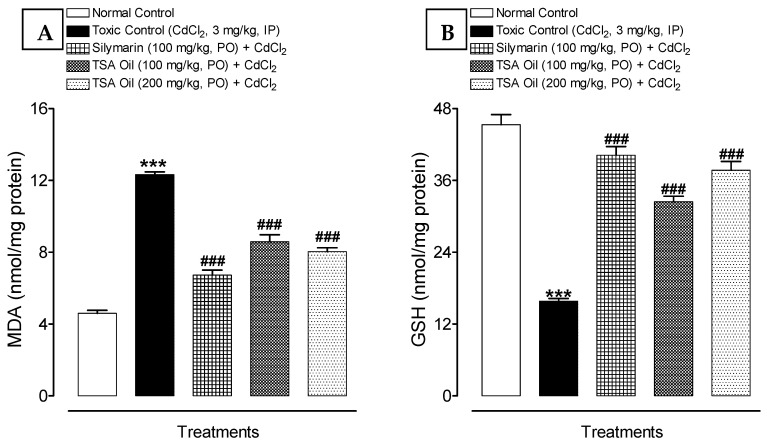
Effect of TSA oil on renal oxidative stress markers (**A**) MDA and (**B**) GSH against Cd-induced renal injury in rats. *** *p* < 0.001, shows the comparison of toxic control with the untreated (normal control) group (unpaired *t*-test); ^###^ *p* < 0.001, shows the comparison of the treated group with toxic control (one-way ANOVA followed by Tukey test). Each bar represents mean ± SEM (*n* = 6).

The renal tissue of rats showed significantly increased lipid peroxidation levels in terms of MDA and reduction of GSH content after Cd administration. In addition, TSA oil treatment at doses of 100 and 200 mg/kg decreases the MDA levels dose-dependently in Cd-intoxicated rats. Further, GSH levels were significantly elevated in the TSA-oil- and silymarin-treated groups compared with the Cd group (Figure 2A,B).

### 2.4. Effects of TSA Oil on p65, NF-κB, iNOS, and Smad2 mRNA Expression

**Figure 3:**
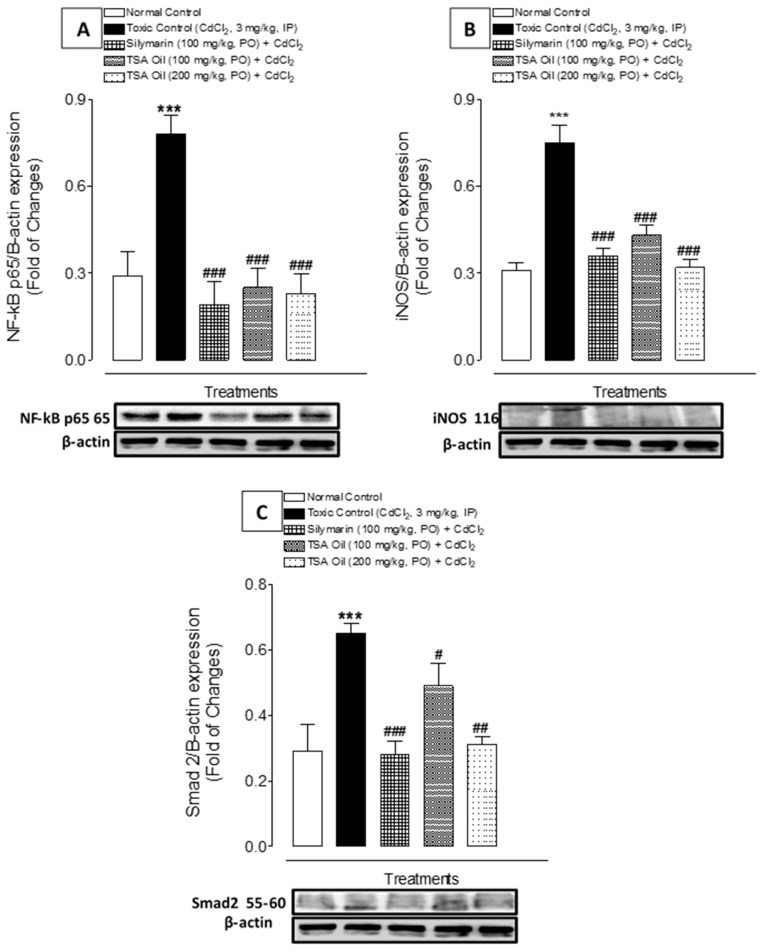
Effect of TSA oil on (**A**) NF-kB, (**B**) iNOS, and (**C**) Smad2 in kidney of Cd-treated rats. *** *p* < 0.001, shows the comparison of toxic control with untreated (normal control) group (unpaired *t*-test); ^#^ *p* < 0.05, ^##^ *p* < 0.01, and ^###^ *p* < 0.001, show the comparison of treated group with toxic control (one-way ANOVA followed by Tukey test). Each bar represents mean ± SEM (*n* = 6).

The changes in the signaling events were assessed by Western blotting. Western blot analysis suggested that Cd exposure significantly (*p* < 0.001) upregulated the NF-κB p65, iNOS, and Smad2 mRNA expression as compared with the reference group. TSA oil treatment significantly downregulated the p65, NF-κB, iNOS, and Smad2 mRNA expression in a dose-dependent manner relative to rats subjected to Cd exposure only (Figure 3A–C).

### 2.5. Effect of TSA Oil on Histopathology

**Figure 4:**
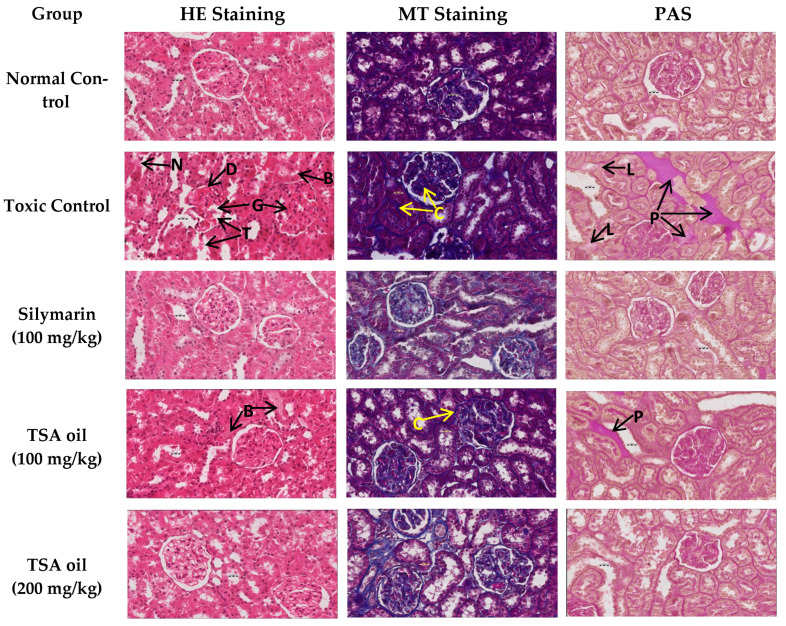
The effect of TSA oil on Cd-intoxicated kidney tissues as assessed by histopathological studies with hematoxylin and eosin (H&E), Masson’s trichrome (MT), and periodic acid–Schiff (PAS) staining. Photomicrographs of toxic group show abnormal glomeruli (G), degenerated (D) and necrotic (N) convoluted tubules, degenerated and almost absent Bowman space. Abnormal convoluted tubules indicate histological disruption (T), blocked by damaged tissue (B) and necrosis. Photomicrograph of MT-stained toxic group shows increased collagenic materials (C). Photomicrograph of PAS staining toxic group shows abnormal deposition of PAS-positive materials (P) and loss of intact basement membrane (L) (magnification 400×; scale bar 20 µm).

Histopathological analysis of the control group rats showed normal kidney histological architecture with normal convoluted tubules, glomerulus, and Bowman capsule with normal space as well as the normal status of collagen fiber and periodic acid–Schiff (PAS)-positive materials such as basement membrane (Figure 4). Photomicrographs of the toxic group show abnormal glomeruli (G), degeneration (D) and necrosis (N) of convoluted tubules, and degenerated and almost absent Bowman space. Abnormal convoluted tubules indicate histological disruption (T), blocked by damaged tissue (B) and necrosis. Photomicrograph of Masson’s trichome (MT)-stained toxic group shows increased collagenic materials (C). Moreover, the photomicrograph of the PAS-stained toxic group shows abnormal deposition of PAS-positive materials (P) that displaced the parenchyma of the kidney; it also shows weakness or loss of intact basement membrane (L). Treatment with TSA oil at a low dose reinstated the normal glomeruli and normal convoluted tubules, although there was still slight suffering from toxic effects in the form of blocking tubules (B) and little increased collagenic material (C), as well as abnormal deposition of PAS-positive materials (P). Treatment with TSA oil at a high dose gave histological pictures of almost normal histological appearance very near to that of the standard treatment (magnification 400× and scale bar 20 µm for all photomicrographs).

## 3. Discussion

In the environment, heavy metals are usually present and are prone to absorption, oxidation, and interference with the fate of cells. Particularly, Cd is a natural industrial compound that causes numerous toxic and hazardous impacts, including nephrotoxicity, on human health [31,32]. Its deposition in various organs causes oxidative stress that weakens the function of the native antioxidant system and, as a result, leads to the development of various severe pathological diseases [33]. The pathway of the Cd-induced toxicity includes the production of reactive oxygen species (ROS), which subsequently causes renal injury [34]. Therefore, it can be assumed that antioxidants may be a good target for the possible therapeutic approach towards Cd-related toxicity [35]. In this report, we studied the promising ameliorative effect of TSA oil on Cd-induced oxidative stress and renal injury using rats.

Serious Cd-induced renal damage may be associated with elevated serum levels of urea, uric acid, and creatinine owing to leakage into the bloodstream [36,37]. In the present study, significantly increased levels of urea, uric acid, and creatinine in serum were also reported after Cd exposure for 7 days, which confirmed the severe renal injury (Figure 1). The observed results corroborate previous studies [19,38]. Administration of TSA oil at both doses (100 and 200 mg/kg, PO) significantly improves renal dysfunction induced by Cd that was supported by the decreased serum levels of urea, uric acid, and creatinine.

In the present study, it has been observed that Cd exposure results in kidney damage by enhancing the lipid peroxidation and disturbing the natural antioxidant system, thus indicating increased oxidative stress. Observed findings suggest that TSA oil could prevent Cd-induced changes in antioxidant-related variables in rats. These findings are in agreement with those of Kawamoto et al. [39], who reported an increased lipid peroxidation after Cd exposure. Earlier studies reported that chronic Cd exposure resulted in diminished nonenzymatic (tissue GSH) antioxidants [40]. Findings from the present study corroborate those of Koyuturk et al. [41], who described reduced renal GSH content in Cd-intoxicated rats. The decreased GSH contents might be due to its consumption in the prevention of lipid peroxidation caused by oxidative stress [41] and detoxification of heavy metals [42,43]. However, our findings disagree with those of Kamiyama et al. [44], who described an elevated level of renal GSH in Cd-treated rats. Lipid peroxidation and antioxidant level in Cd-treated rats were significantly modulated with TSA oil administration, indicating a decrease in ROS, a rebalancing of the antioxidant defense system, or both. These findings corroborate those of an earlier study that described the ameliorative effect of thymoquinone against chemical-induced renal injury [45].

Apoptosis occurs in tissues and varies from necrosis under certain functional disorders [46]. Apoptosis is one of the principal characteristics of Cd-related kidney injury. Earlier, it was described that Cd toxicity causes apoptosis in proximal tubules through stimulation of the NF-kB pathway, which contributes to renal dysfunction [47,48]. In this study also, an increased NF-kBp65 protein expression was observed in Cd-treated rats. However, administration of TSA oil in Cd-treated rats substantially restored the NF-kBp65 protein expression. The observed findings are in agreement with the previous findings [18,19]. Therefore, NF-kB inhibition in Cd-intoxicated rats has evidenced the anti-inflammatory ability of TSA oil.

Inducible nitric oxide synthase (iNOS) is highly expressed in inflammatory conditions and infections. Thus, it is a vital component of the adaptive response of the host to noxious stimuli. It was reported earlier that Cd-induced renal injury is mediated by nitric oxide synthesis by iNOS stimulation [49]. In this study also, Cd exposure was found to cause increased iNOS expression (Figure 3B), while TSA oil at both doses could significantly reverse iNOS expression in Cd-intoxicated rats.

Previous literature reported that Cd is one of the most toxic heavy metals and is mainly absorbed by the proximal tubules and accumulated mostly in the renal cortex, which leads to lesions in proximal convoluted tubules [50]. These findings corroborate those of the present study. Major kidney damage was verified by the presence of significant damage to glomeruli in Cd-treated rats. These findings corroborate those of Damek-Poprawa and Sawicka-Kapusta [51], who reported atrophy of glomerular capillaries and necrosis of proximal tubules. The findings of kidney function markers and oxidative stress markers—where TSA oil ameliorated Cd-induced degenerative changes and improved kidney function owing to its possible antioxidant and anti-inflammatory effects—have been confirmed by histopathological observations.

## 4. Materials and Methods

### 4.1. Chemicals and Reagents

Cadmium chloride (CaCl_2_) was obtained from Sigma Chemicals Co. (St. Louis, MO, USA). Kidney function diagnostics kits were obtained from Crescent Diagnostics (Jeddah, KSA). Antibodies (primary as well as secondary) were procured from Santa Cruz (Dallas, TX, USA). Chemicals used were of analytical grade and high quality that did not require further purification.

### 4.2. Plant Material and Extraction

Fresh aerial parts of *T. serrulatus* Hochst. ex Benth were collected from the Amba Alaje mountain area, South Tigray, Ethiopia. The plant material was authenticated by a botanist, Dr. Getinet Masresha, Department of Biology, University of Gondar, and the specimen was deposited at the herbarium of the university (TH-001/2011).

The fresh aerial parts were cut into small pieces and subjected to hydrodistillation for 3 h using a Clevenger apparatus. Hydrodistillation was performed 11 times until a sufficient amount was collected. The obtained essential oil was dried using anhydrous sodium sulfate and stored in a tightly closed container at 4 °C until further use [26]. The calculated essential oil yield was expressed in percentage (% *v*/*w*), based on the weight of the fresh plant material.

### 4.3. Animals

Thirty male albino rats weighing 180–220 g were obtained from Animal House, College of Pharmacy, Prince Sattam Bin Abdulaziz University (PSAU), KSA. Rats were acclimatized for 1 week and maintained under 12 h light/dark cycles with standard laboratory facilities. During the experimental and acclimatized period, animals were fed with a pellet diet and free access to water. Instructions and Guidelines provided by the animal care unit, PSAU, KSA, were followed when performing all of the experiments. The protocol was preapproved by Bio-Ethical Research Committee (BERC), PSAU (BERC-004-12-19).

### 4.4. Experimental Design

For the assessment of the nephroprotective effect of TSA oil against Cd-induced toxicity, rats were randomly allocated into five groups (*n* = 6). Group 1 (normal control) received physiological salt solution (0.9% NaCl) daily for 7 days. Group 2 (toxic control) received cadmium chloride (3 mg/Kg, IP, 7 days). Group 3 (positive control) was coadministered CdCl_2_ and silymarin (100 mg/kg, PO) for 7 consecutive days. Groups 4 and 5 were coadministered CdCl_2_ and TSA oil at doses of 100 and 200 mg/Kg (PO, 7 days), respectively. The dose of CdCl_2_ was selected based on previously reported literature [19,52].

After 24 h of treatment, all the male rats were anesthetized with a small amount of diethyl ether to collect the blood samples from retro-orbital plexus followed by centrifugation, and the separation of serum was performed and stored at −20 °C until further use for the determination of kidney function markers (like uric acid, creatinine, and urea). After successful serum collection, both the kidneys were isolated from all the rats. The left kidney was immediately stored at −80 °C until the further analysis of oxidative stress markers (MDA and GSH) and Western blot analysis. Meanwhile, the right kidney was stored for histopathological studies.

### 4.5. Determination of Kidney Function Biomarkers

Biomarkers of kidney function, i.e., uric acid, urea, and creatinine, were evaluated by using specific commercial kits as per the methods mentioned in manufacturers’ protocols.

### 4.6. Determination of Oxidative Stress Markers in Kidney

Tissues were homogenized and minced (10% *w*/*v*) in ice-cold 0.1 M phosphate buffer at pH 7.4, and the solution was centrifuged for 30 min at 12,000× *g* and 4 °C. The homogenate obtained was used for the estimation of GSH and MDA levels.

MDA lipid peroxidation marker was estimated by using a previously described method [53]. Briefly, 0.25 mL of homogenate was incubated at a temperature of 37 ℃ for 1 h in a metabolic shaker. After incubation, 0.5 mL of 0.67% thiobarbituric acid (TBA) and 0.5 mL of 5% (*w*/*v*) chilled trichloroacetic acid (TCA) was added, followed by centrifugation (1000× *g*, 15 min). Thereafter, the supernatant was kept in a boiling water bath for 10 min. The absorbance developed was measured at 535 nm, i.e., pink color observed.

For GSH, the Jollow et al. [54] method was followed. Briefly, after precipitation of 1 mL PMS with 1 mL of sulfosalicylic acid (4%), the test samples were incubated (4 °C, 1 h), followed by centrifugation (1200× *g*, 15 min, 4 °C). The assay mixture contained supernatant (0.1 mL), phosphate buffer (0.1 M, pH 7.4) (1.7 mL), and dithio-bis-2-nitrobenzoic acid (DTNB) (0.4% in phosphate buffer, 0.1 M, pH 7.4) (0.2 mL) in a total volume of 2.0 mL. The absorbance of samples was analyzed at 412 nm within 5 min after the addition of DTNB to the reaction mixtures.

### 4.7. Western Blot Technique

Western blot analysis and protein extraction were performed as mentioned in the previous study [55]. Kidney tissues were minced and the homogenate was prepared in a protease inhibitor mixture and cold protein lysis buffer [55]. To isolate the total proteins, the tissue lysates were preserved in ice for 60 min, with alternative vortexing (after 10 min), continued with the centrifugation at 12,000× *g* (4 °C, 10 min). The method of Lowry et al. [56] was followed to determine the total protein. For Western blot analysis, protein (25–50 µg) was briefly isolated from each group and shifted to nitrocellulose membranes obtained from Bio-Rad USA. Immediately blocking of protein blots was done at 4 °C (for 24 h); after that, incubation with primary antibodies against NF-κB p65, iNOS, and Smad2 and peroxidase-conjugated secondary antibodies at room temperature was performed. The proteins were analyzed with the help of a chemiluminescence detection kit (GE Health Care, Mississauga, Canada). Protein band intensity was normalized to beta-actin bands using the ImageJ (NIH, Bethesda, USA). Images were captured with a C-Digit chemiluminescent Western blot scanner obtained from LI-COR, USA.

### 4.8. Histopathological Analysis

Right kidney sample tissues fixed in 10% neutral formalin were managed in a tissue processing machine (ASP300s, Leica Biosystems, IL, USA); each sample was embedded in paraffin wax and sliced into 4–5-µm-thick sections. Three sections of each group were chosen and stained with hematoxylin and eosin (H&E) dye, periodic acid–Schiff (PAS) stain, and Masson’s trichrome (MT) stain [57]. All the sections of tissue were analyzed using a microscope (Olympus BX 52) for histopathological description which was recorded by a histopathologist, blinded to the experimental groups. Olympus DP21 camera fixed over the microscope was used for taking photographs.

### 4.9. Statistical Analysis

Values were expressed as mean ± SEM. One-way ANOVA analysis using post hoc Tukey’s test was used to measure the significance of biochemical data of the different groups. Differences were measured significant at *p* values < 0.05 relative to the normal control or toxic control group. The statistical investigation was achieved using GraphPad Prism v. 4.0 (USA).

## 5. Conclusions

This study concludes that the essential oil of *T. serrulatus* ameliorated Cd-induced renal impairment, which was possibly mediated by improvement in the altered biochemical and oxidative stress parameters in addition to improvement in the histological structures. Furthermore, *T. serrulatus* might be an appropriate agent to be developed in the future for renal protection and kidney-related disorders.

## Data Availability

Data sharing not applicable.

## Sample Availability

Samples of the TSA essential oil are available from the authors.
